# Setting up a nurse-led model of care for management of hypertension and diabetes mellitus in a high HIV prevalence context in rural Zimbabwe: a descriptive study

**DOI:** 10.1186/s12913-020-05351-x

**Published:** 2020-06-01

**Authors:** Marthe Frieden, Blessing Zamba, Nisbert Mukumbi, Patron T. Mafaune, Brian Makumbe, Elizabeth Irungu, Virginia Moneti, Petros Isaakidis, Daniela Garone, Madhu Prasai

**Affiliations:** 1 Médecins Sans Frontières, 7 Bougainvillea close, Palmerstone, Mutare, Zimbabwe; 2 Ministry of Health and Child Care, Manicaland, Zimbabwe; 3 MSF Southern Africa Medical Unit, Cape town, South Africa; 4 Médecins Sans Frontières, Brussels, Belgium

**Keywords:** Nurse-led, Hypertension, Diabetes mellitus, Mentoring, Non-communicable diseases, Primary-health-care

## Abstract

**Background:**

In the light of the increasing burden of non-communicable diseases (NCDs) on health systems in low- and middle-income countries, particularly in Sub-Saharan Africa, context-adapted, cost-effective service delivery models are now required as a matter of urgency. We describe the experience of setting up and organising a nurse-led Diabetes Mellitus (DM) and Hypertension (HTN) model of care in rural Zimbabwe, a low-income country with unique socio-economic challenges and a dual disease burden of HIV and NCDs.

**Methods:**

Mirroring the HIV experience, we designed a conceptual framework with 9 key enablers: decentralization of services, integration of care, simplification of management guidelines, mentoring and task-sharing, provision of affordable medicines, quality assured laboratory support, patient empowerment, a dedicated monitoring and evaluation system, and a robust referral system. We selected 9 primary health care clinics (PHC) and two hospitals in Chipinge district and integrated DM and HTN either into the general out-patient department, pre-existing HIV clinics, or an integrated chronic care clinic (ICCC). We provided structured intensive mentoring for staff, using simplified protocols, and disease-specific education for patients. Free medication with differentiated periodic refills and regular monitoring with point of care (POC) glycosylated haemoglobin (HbA1c) were provided.

**Results:**

Nurses in 7 PHC facilities and one hospital developed sufficient knowledge and skills to diagnose, initiate treatment and monitor DM and HTN patients, and 3094 patients were registered in the programme (188 with DM only, 2473 with HTN only, 433 with both DM and HTN). Major lessons learned from our experience include: the value of POC devices in the management of diabetes; the pressure on services of the added caseload, exacerbated by the availability of free medications in supported health facilities; and the importance of leadership in the successful implementation of care in health facilities.

**Conclusion:**

Our experience demonstrates a model for nurse-led decentralized integrated DM and HTN care in a high HIV prevalence rural, low-income context. Developing a context-adapted efficient model of care is a dynamic process. We present our lessons learned with the intention of sharing experience which may be of value to other public health programme managers.

## Background

The epidemic of Non Communicable Diseases (NCDs) in Sub-Saharan Africa (SSA), mainly cardiovascular diseases (CVD), cancer, diabetes mellitus (DM) and chronic lung disease, and its impact on existing health systems is increasingly being reported [1, 2]. The Global Burden of Disease study identified hypertension (HTN) and DM as the leading risk factors for early death and disability globally [1]. In Zimbabwe, the overall pooled prevalence of HTN was 30% between 1997 and 2010 [3], while the prevalence of DM has been increasing significantly over the past three decades from 0.44% in 1980 to 5.7% in 2013 [4]. Zimbabwe also has a high HIV prevalence of 14% among adults 15 to 64 years [5]. The overlap between HIV and NCDs is substantial: HIV and its treatment increase the risk of developing HTN and DM [6]; the success of Antiretroviral Therapy (ART) programmes means that increasing numbers of people are surviving long enough to develop NCDs. 14% of people living with HIV (PLHIV) in Zimbabwe currently suffer from at least one NCD, and this figure is expected to double by 2035 [7].

The ART treatment programme is an example of a successful model of chronic disease care in Zimbabwe where the health system is primarily oriented towards management of acute infections and maternal and child health. Various models of DM and HTN care exist worldwide, with gold standard management guidelines for DM and HTN based on western experience, where multidisciplinary teams offer specialized, resource-intensive care. These are poorly adapted for low- and middle-income countries (LMIC), where nurses are the frontline workers attending to patients as they enter the health system at primary health care level (PHC). Models of care for DM and HTN based on task-shifting to non-physician clinicians and decentralisation to primary care have been successfully demonstrated in other countries in SSA [8], but not to our knowledge in Zimbabwe.

Médecins Sans Frontières (MSF) has been supporting the Ministry of Health and Child Care (MOH) in Zimbabwe since 2004 to roll out ART to PHC facilities. In 2016, MSF together with MOH designed and implemented a context-adapted model of care to address the burden of DM and HTN in Manicaland, leveraging lessons learned from the successful ART scale up programme. Although the principle of adapting lessons learned from the HIV experience to NCD programmes is widely recognized [8], there are significant knowledge gaps with regard to practical implementation of the management of NCDs including policies, protocol simplification and standardization, training of staff, and supply of affordable medications and laboratory consumables [9]. We leveraged our programmatic experience in HIV to implement an nurse-led model of DM and HTN care, decentralised to primary health level, in a rural district of Zimbabwe.

The aim of this paper is to describe the components of our model of care, with particular emphasis on the evolution of the programme as it was implemented and the lessons learned from our experience which may be of value to other NCD programmes in SSA.

## Methods

### Design

This is a programmatic description of a model of care for DM and HTN services.

### General study setting

The study took place in Chipinge rural district, one of the seven districts of Manicaland in Zimbabwe. The economy is based on subsistence and commercial farming, with low incomes per household [10] . It has a population of over 300,000 [11] served by 51 health facilities. The dirt road network makes some health facilities hard to reach, especially during the rainy season. Chipinge District Hospital (CDH) is the major referral hospital for the North and St Peter’s Mission Hospital (SPMH) for the South. Reliable figures on DM and HTN prevalence in Chipinge district have not been documented.

### The MOH/MSF Chipinge DM and HTN programme

MOH/MSF began activities in July 2016 in 11 health facilities, offering care for patients with DM and HTN. An MSF mentoring team provided structured teaching sessions and hands-on clinical training to MOH staff, who performed consultations. No NCD-specific guidelines were available in Zimbabwe, therefore MOH/MSF developed simplified context-adapted clinical protocols, training materials and patient literacy tools. MSF supported MoH to meet the cost of medications and laboratory consumables. Patients who attended health facilities for DM and HTN care were registered in the programme.

### Conceptual framework and key enablers for NCD programmes

**Fig. 1:**
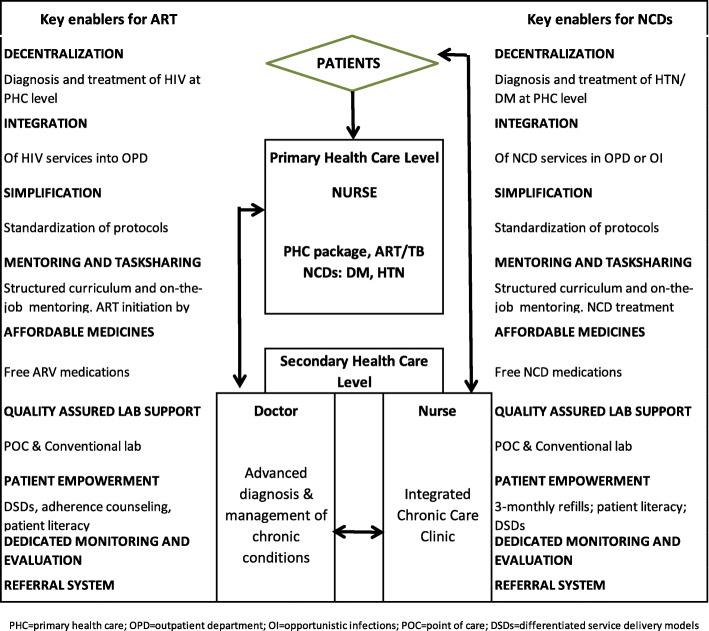
Conceptual framework

Our programme design was based on a conceptual framework (Fig. 1) developed by the authors by drawing from MSF’s experience on HIV care in SSA including Zimbabwe. We were also inspired by various publications describing successful strategies used in delivering HIV care across the entire health pyramid [12–14]. The mid-section of the framework illustrates the health system. Patients within the community can access their PHC facility for acute or chronic care, and maternal and child health services, where they are attended to by qualified nurses. Where the condition requires expertise, patients are referred to secondary or tertiary levels. Once patients are stable, they are then referred back through the various levels down to PHC level. The left-hand column highlights the 9 strategic key-enablers of a successful ART programme while the right-hand column mirrors the same strategies for the NCD programme**.**

### Technical and treatment choices

We used automated sphygmomanometers to measure blood pressure (BP) and diagnosed HTN if two out of three readings were ≥ 140/90 mmHg on 3 separate visits (or 2 separate occasions if BP ≥ 180/110 mmHg). Any adult presenting to a health facility in Zimbabwe receives a blood pressure check, and among HIV patients annual screening for HTN is recommended [15]. For DM we adopted a health facility-based opportunistic screening approach according to risk factors such as family history, presence of HTN, HIV, active TB, obesity, CVD or Chronic Kidney Disease (CKD). Diagnosis of DM was based upon a combination of two tests: glycosylated haemoglobin (HbA1c) ≥6.5% and a random blood sugar (RBS) ≥11.1 mmol/L, or a fasting blood sugar (FBS) ≥7 mmol/L. In the presence of severe symptoms of hyperglycaemia, a single high reading of blood glucose confirmed diagnosis (see Table 1).

**Table 1:** Diagnosis of DM: test combinations required

Test 1	Test 2
FBS ≥ 7.0 mmol/L	AND HbA1c ≥ 6.5%
RBS ≥ 11.1	AND HbA1c ≥ 6.5%
FBS ≥ 7.0 mmol/L OR RBS ≥ 11.1 mmol/L AND symptoms of hyperglycaemia	Not applicable No second test required

Those with self-reported conditions were rescreened to confirm the diagnosis, if there was insufficient clinical or documentary evidence to support the accuracy of the initial diagnosis. For both conditions, active screening in the community was avoided as this has been shown not to be cost-effective [8, 16], and due to concerns that it might overload health facilities with patients.

The treatment initiation threshold for patients with HTN-only was set at BP ≥160/100 mmHg in line with the WHO/ISH risk stratification [17] and standard MSF protocols. For patients with additional risk factors such as diabetes, known CVD or CKD, a lower threshold was chosen (BP ≥140/90 mmHg). The initial treatment target was defined as BP < 140/90 mmHg for patients < 65 years, and BP < 150/90 for patients ≥65 years. Subsequently we simplified protocols, and set a single target of 140/90 for all age-groups.

HbA1c targets for diabetes were initially defined as < 8% for < 65 years, and < 9% for ≥65 years. A single target of < 7% for low risk patients independently of age was subsequently chosen within a rationale of simplification. A target of < 8% was set for high risk patients (elderly, history of severe hypoglycaemia, multiple comorbidities, long-standing diabetes, limited life expectancy or advanced chronic diabetic complications).

## Results

Between July 2016 and June 2019, 3094 patients were registered in the programme (188 with DM only, 2473 with HTN only, 433 with both DM and HTN). We describe below our experience of setting up the programme along the lines of our conceptual framework of enablers. Table 2 below summarizes the enablers, key components and lessons learned.

**Table 2:** Enablers, key components of each enabler, and lessons learned during programme implementation

Enabler	Key components	Lessons learned
1. Decentralisation	Service set-up and empowerment of nurses to diagnose and manage DM and HTN	Nurse-led services fully operational in 7/9 PHC facilities and 1 hospital. Spread patient visits throughout the week to manage workload. Difficult to ensure regular access of mentoring teams to remote sites
2. Integration	Two models emerged for NCD integration: either in general OPD or with HIV services.	What works best depends on individual circumstances of each health facility Choose the best fit rather than imposing a ‘one size fits all’ model
3. Simplification	Context-adapted SOPs Experience-based fine-tuning of clinical treatment	Guideline development is a dynamic process requiring internal and external expertise, responding to user feedback
4. Mentoring and task sharing	Multidisciplinary MSF mentoring teams and MoH mentees Mentoring curriculum comprising clinical and programmatic knowledge On-the-job support by mentor. Competence dashboard to monitor progress	Major limitations: Long travel distance for mentors, HR shortages limiting the availability of dedicated key staff for regular mentoring, high patient volume Requirement for ownership and leadership in health facilities
5. Affordable medicines	Rational medication choices Free medications subsidised by MSF Advocacy to improve MoH medication supply to health facilities	Free medications improved access to care for patients Reliable MSF-supported supply allowed spacing of patient appointments ‘Pull factor’ of free medications increased programme demand
6. Quality assured lab support	Use of pre-existing laboratory systems and equipment Introduction of POC machines for glucose and creatinine measurement External Quality Assurance system for HbA1c	Problems with transport of samples to central laboratory overcome by on-site POC machines Trained and motivated laboratory staff required to produce quality results when conventional machines are used
7. Patient empowerment	Individual and group literacy sessions for DM and HTN	Successful implementation of DM and HTN literacy sessions
8. Dedicated Monitoring and Evaluation	Design and implementation of patient records for DM and HTN consultations Data collection in electronic database and quarterly analysis Development of standard indicators	Simplification of patient records improved completeness and quality of data Dedicated M&E system improved resource quantification
9. Referral system	Development of referral criteria to higher level for complex patients	On- and off-site decision support by doctors

### Decentralization

Currently, according to the Zimbabwean national policy, diagnosis of DM and HTN is under the responsibility of doctors at hospital level, while refills of selected NCD medications can be given at PHC level. The programme did not aim for coverage of the entire health district, but rather to introduce DM and HTN care at a selection of 11 PHC sites in line with available resources. The site selection process was done together by MOH and MSF. First, we excluded clinics in urban settings and hospitals other than the referral sites. Then, using a quantitative and qualitative evaluation process, rural clinics were scored and ranked according to a set of inclusion and exclusion criteria described in Table 3.

**Table 3:** Criteria for site selection in Chipinge District

Inclusion criteria	Exclusion criteria
Larger size of population served	Number of nurses < 2 per clinic
Larger size of ART cohort	Driving time from district capital to health facility > 90 min (one-way)
Higher number of documented cases of NCDs	High turnover of human resources
Higher number of nurses/clinics	Proximity to other possible selected clinics
Stronger recommendation of MOH	Receiving support from other NGOs

At the end of 2018, two PHC sites struggling to adopt the services were dropped from the pilot, while two with satisfactory results but a very remote location were handed over to MOH .

### Integration

In the Zimbabwean ART programme, some health facilities have vertical services exclusively for HIV positive patients, while in other sites care for HIV patients is integrated within the day-to-day general outpatient department (OPD). Thus in our programme, DM and HTN were either managed in an integrated chronic care clinic (ICCC) where patients with HIV and/or NCDs receive care in the same service, or merged with the general OPD [18]. Our aim was to search for the ‘best fit’ model for service integration for each facility. Regardless of which model was used, when PLHIV had DM or HTN, files would be merged and appointment dates synchronized so that both conditions could be treated at a single visit.

Two out of 9 PHC facilities with existing separate HIV care added and merged DM and HTN management. In 7 out of 9 health facilities the general OPD was the underlying platform for integration. This yielded acceptable results to both staff and patients without compromising existing services. One of these facilities subsequently attempted to move HIV and NCD patients away from the OPD into an ICCC. However, this stretched pre-existing human resource shortages and the coping threshold was reached when the cohort increased in size forcing them to revert to integrating HIV and NCDs into the general OPD.

At the two secondary care facilities, HIV and OPD services operated independently in separate departments at the inception of the pilot. Our initial approach was to introduce DM and HTN services within the general OPD for HIV negative patients, while separately supporting the HIV department to diagnose and manage DM and HTN among their existing ART cohort. However, at SPMH an overwhelming number of DM and HTN cases began to compromise OPD services. Given the smooth functioning of the HIV department, we decided that this would be a better site to host all DM and HTN care. By building additional clinic infrastructure, adding human resources and optimizing the organization of existing services within the HIV department, we established a well flourishing integrated chronic care clinic (ICCC). CDH continued to operate in two different sites according to HIV status, due to HR challenges and infrastructural constraints. Organisation of DM and HTN care in this hospital did not achieve the same level of service delivery as at SPMH.

### Simplification

Learning from the ART programme which used simplified clinical guidelines and treatment algorithms to ensure safe use at PHC, we developed standard operation procedures (SOPs) for clinical management (see Additional file 1), adopted from evidence-based international guidelines, MSF guidelines and experience from projects elsewhere in SSA, aligned with the national medicine formulary [19]. Context adaptation was a dynamic process involving regular reviews with technical support from internal MSF and external specialists, and feedback from the clinicians using the protocols and patients.

An example of simplification is with regard to treatment targets for HTN. Initial targets were looser for elderly patients (age > 65 years) in order to reduce the risk of iatrogenic hypotension. Subsequently, as we observed poor target achievements overall with clinical inertia being a possible significant factor, we simplified protocols by choosing a single target of 140/90 for all age-groups.

### Mentorship and task-shifting

We adopted a step-wise on-the-job mentoring approach which emphasized knowledge, practical skills and behaviour. The mentors were provided by MSF in three categories: nurses, pharmacy technicians and doctors. The nurses were qualified Registered General Nurses (RGN, three-year diploma) trained in mentorship, with practical experience in mentoring HIV care. The pharmacy technicians had a four-year diploma training in dispensing and pharmacy management. The doctors were general practitioners with significant experience in chronic disease management. All mentors underwent training in DM and HTN care using context-adapted guidelines and on-the job training by MSF supervisors. Mentees from the MOH comprised of RGNs, Primary Care Nurses (18 months training), nurse aides and primary counsellors (lay cadres with ordinary level training and a 6-month training on counselling HIV patients). A mentoring curriculum on organisation of services and DM and HTN care was developed and linked to an evaluation grid to score competencies (the competency dashboard). Mentees would receive an initial theoretical training (1 day), then, according to an established schedule, two mentoring teams visited the health facilities every 1 to 2 weeks. This on-the-job mentoring cycle was intended for 3 months, after which mentees would graduate and provide DM and HTN care with periodic on-site and off-site decision support (task-sharing). In certain instances, an extra nurse would be added to the mentoring team to free the MOH nurse undergoing mentorship from her/his usual tasks. Mentoring involved (a) on-site group meetings with all MOH staff with case discussions and lectures on related topics, (b) side-by-side clinical decision support and/or counselling, (c) practical demonstrations of efficient service organisation, including organisation of patient flow and spacing of appointments, and pharmacy management practices. Review meetings were organised in clusters twice a year to analyse performance for a group of health facilities, and to exchange experience.

This mentorship approach allowed a small, mobile team of mentors to set up services in multiple sites simultaneously, thereby accelerating the provision of DM and HTN services and standardising practices across 11 sites. Three sites (one hospital and two PHC clinics) with the highest potential were given a more intensive mentorship schedule and developed as model sites. When sufficient technical capacity was built, patient appointments were spread throughout the week, rather than being clustered on the day of the mentorship visits, and mentorship time was decreased.

Seven out of 9 PHC facilities and one hospital achieved sufficient competency-dashboard scores to diagnose, initiate treatment and monitor DM and HTN patients. However, even when dashboard indicators were achieved, the desired knowledge and competencies were attained over a longer period than expected. Challenges for mentors were long travelling time which decreased the daily mentorship coverage or high DM and HTN patient volume on arrival which limited the mentorship time. As noted above, two very remote sites were handed over earlier than planned after having achieved basic skills, in order to free up mentoring time. Due to general human resource (HR) shortages, MOH faced difficulties in freeing up dedicated core staff for regular mentoring, preventing some nurses from completing a full mentoring cycle. To address the problem of staff shortages and high workload, we hired additional staff on short contracts as a temporary solution. We felt a major reason for failure to achieve mentorship targets was lack of clear programme leadership and poor staff ownership of their additional duties. We noted that when good leaders emerged and staff were motivated and willing to be mentored, positive results were achieved, and conversely.

### Affordable medicines

To standardise treatment, medications were chosen, titrated upwards and different classes added stepwise in consecutive consultations based on clinical and laboratory results. Choice of medications was a compromise between effectiveness, availability, affordability and user friendliness informed by the WHO essential drug list [20], the Zimbabwe national formulary [19] and international guidelines [21, 22]. Before the start of the pilot, NCD medication supply to health facilities was inadequate and inconsistent, with frequent stock outs. Patients received less than a month’s supply and had to come more frequently to top up their prescriptions, or even had to buy medications privately. Patients reported taking suboptimal dosages, or not taking treatment at all until it became available. Thus for this programme, medicines and laboratory reagents were largely provided by MSF, and based on MSF principles, they were given for free to all patients registered. The reliable medication supply allowed us to offer three-monthly medication refills for stable patients. A component of advocacy was embedded in the pilot to lobby at facility, district, provincial, national and international levels for resource mobilization for DM/HTN medicines.

However, providing free medications in our pilot proved a pull factor attracting patients from neighbouring districts and provinces. The worsening economic situation in late 2018 further exacerbated this problem as patients who were previously able to afford to buy their own medications were no longer able to do so and turned to MSF-supported clinics for subsidised drugs. This meant that the MoH was not able to increase its responsibility for medication supply, maintaining dependence on MSF.

### Equipment and quality assured laboratory support

Our programme took advantage of existing MOH staff, infrastructure and equipment. Existing conventional laboratory-based biochemistry machines were used to measure creatinine, blood glucose and HbA1C at hospital level. Despite reinforcement, the existing sample transport system proved to be inefficient as sample transport time was as long as 2 to 3 days from the farthest clinics to the hospital laboratory, interfering with the quality of the samples. The laboratories became overloaded and the result turnaround time was long. We then opted for handheld glucometers for diagnosis of DM, and in 2018 introduced point of care (POC) machines for HbA1C (Fine Care®) and creatinine (Novastart®) measurement. All HbA1C testing platforms were enrolled in a monthly External Quality Assurance (EQA) scheme. Table 4 below chronicles the simplification process of the SOPs for diagnosis of DM.

**Table 4:** Simplification process of SOPs for the diagnosis of DM

	2016	2017	2018	2019
Diagnostic approach At site level	No diagnostic devices used at site level	FBS/RBS on hand-held glucometer	FBS/RBS on hand-held glucometer	FBS/RBS on hand-held glucometer And HbA1c on Point of care platform to confirm
Conventional laboratory (hospital based)	Lab-based FBG	Confirm with lab-based FBG	Not applicable	Not applicable
	Lab-based HbA1c to confirm	Lab-based HbA1c for final diagnosis	Lab-based A1c to confirm	Not applicable
Rationale	Dependency on sample transport (ST) and stability Lab overwhelmed Delays in result reception	Same dependency on ST Lab workload improved Still delays in result reception	Same dependency on ST Lab workload improved. Still delays in result r*e*ception Need for motivated HR to follow quality control procedures for HbA1c	Increased autonomy at PHC Faster decision making

We found that the use of conventional laboratory machines for HbA1c needed motivated human resources to follow the quality control procedures required. Performance improved after the introduction of POC devices. DM monitoring with HbA1c is a fairly new concept in SSA where there may be additional sources of error due to haemoglobinopathies or malaria [9], or high HIV prevalence [23, 24]. However, HbA1c measurement was a game-changer against the inconvenience of repeated blood sugar measurements and the poor correlation of these with good glycaemic control.

### Patient empowerment

This concept involved enabling patients to acquire the knowledge and skills to understand and take responsibility for their own health. Individual and group counselling sessions for DM and HTN emphasised knowledge about glycaemic and blood pressure (BP) control. An active decision not to prioritise defaulter tracing was taken as resources were scarce and we considered there was no public health danger, contrary to contagious diseases such as HIV or TB. In the long run, we aim to differentiate services according to the needs of specific patient subgroups i.e. differentiated service delivery (DSDs) models. At the time of writing this report, DSDs are emerging at community and health facility level.

### Dedicated monitoring and evaluation (M&E)

Before the programme started, there were no individual patient files for NCD patients.

Health facilities used improvised registers, which did not allow recording of information on follow up and treatment outcomes. There was likely substantial under-diagnosing and underreporting of NCDs.

We designed medical records inspired by the ART patient files and provided them at all sites. The files accommodated both identification numbers for HIV and DM/HTN to enable health workers to identify patients with co-existing conditions and to synchronize appointments. A set of indicators for monitoring and evaluation, following the standard cohort approach used in HIV/TB control programmes, were defined to measure service provision, case-enrolment, follow up, treatment results and retention in care (see Additional file 2). Data from patient clinical records were entered onsite or offsite into an electronic database by trained data encoders and were analysed during quarterly review meetings with mentees and managers for use in programmatic decision making. Further support for data evaluation was provided intermittently by MSF technical referents.

With specific and detailed patient records in place, nurses were able to provide improved longitudinal follow up for patients as well as quantifying DM and HTN service demand and medication needs for the programme. In some sites incomplete data was a challenge, and this increased as the cohort sizes grew. A simplified chronic patient card with only key parameters for clinical decision-making was therefore implemented in late 2018. We observed that minimizing the number of variables and storing the patient records close to the consultation area increased completeness of data.

### Referral system

The focus of this programme was on empowering nurses to manage DM and HTN, thereby minimising referrals to secondary care. Where the management of complex cases exceeded the limits of the care provider, context-adapted criteria were developed to identify these patients in a timely way for consultation by a medical doctor on or off site.

## Discussion

Nurse-led and PHC models of NCD care have been successfully implemented throughout SSA [25–29] and chronic care experience is often drawn from the ART scale up [30]. Our programme is distinguished by its conceptual framework of 9 ‘enablers’, also inspired by HIV experience, using the principles of simplification of the clinical and programmatic package and integration of NCD care alongside other PHC activities. We demonstrated two potential models for integrating DM/HTN care: inclusion as part of the general OPD, and merging together with HIV care in a special integrated chronic care clinic. The driving force for the set-up of our programme was a mentorship approach, whereby light, mobile teams of multidisciplinary mentors provide on the job training to MoH mentees through a mixture of formal structured teaching, clinical supervision and programmatic advice. We also developed a system for monitoring and evaluation of the DM and HTN programme, consisting of patient records adapted for longitudinal follow-up, a system of data collection and standardised indicators. The major barriers to successful implementation were: limitations in HR and infrastructure; pressure on services, particularly due to the attraction of free medications provided by MSF and failure to space clinic appointments throughout the working week; and lack of leadership and ownership by MOH staff in health facilities.

In our model, we set up DM and HTN services at PHC level from the outset, linked to 2 referral hospitals, which is in contrast to Malawi where integrated chronic care clinics were set up first at two hospitals [18], followed by decentralisation of services to 11 health centres due to high defaulter rates and slow enrolment growth. Downstream referral from secondary to primary care has also been used in HIV care [31, 32]. We believe that DM and HTN care should be offered primarily at PHC level as this decongests secondary care and improves access for patients. Multiple integration models have been documented at PHC level, most commonly through merging HIV and NCDs together in specialist clinics [12, 18, 33], whereas in our experience when we allowed facilities to follow their natural evolution, this most frequently resulted in the merging of DM and HTN consultations into the general OPD. Our observation that overcoming infrastructural constraints was the key factor in the success of our ICCC model is supported by other studies [12, 34–36]. One model does not fit all sites, and flexibility and context-adaptation are needed.

In the existing literature, mentorship and coaching interventions, as components of health system strengthening, have been shown to be effective in improving knowledge and clinical practice skills [28, 37], using the key elements of clinical mentorship: local experienced mentors, standardised protocols for consultation and referral of patients, on-site and off-site decision support [38]. Our step-wise on-the-job training emphasising knowledge, practical skills and behaviour and including programmatic instruction to improve organisation of services, is a broader approach than most in-service trainings [39] and remains relatively unusual in SSA, with comparable approaches applied in Rwanda [28] and Kenya [40]. The main implementation challenges we encountered, namely time lost in travelling to mentorship sites, reduced mentorship time due to high workload, and lack of sufficient HR to free up mentees for training, were also found in other mentoring programmes in SSA [37].

Given that one hospital and two PHC facilities were not able to provide nurse-led DM and HTN services autonomously, it is important to discuss reasons for the failure to implement our programme model. The contribution of lack of leadership and ownership by staff in health facilities which we noted has been reported elsewhere. Resistance to take up NCD care, perceived as additional work, has been highlighted in India [41] and in Malawi clear leadership and staff ownership was a key to the success of the project [18]. We recommend that policy makers and managers invest time and resources in identifying responsible leaders and motivating staff at all levels for NCD care. During site-selection willingness of the staff to be mentored might also need to be considered. Managers should guide the staff towards rationalizing the overall workload and restructuring workers’ schedules to accommodate NCD-related work [41].

The second major impediment we encountered was immediate overwhelming demand in some sites through attracting patients from outside the intended catchment area, primarily due to access to free medications in our programme, exacerbated by the worsening economic situation in late 2018. Labhardt et al. in Cameroon offered a decentralized model similar to ours in almost all clinics (69/75) in 8 districts [27], contrary to our choice not to aim for full coverage of the health district, but instead of overcrowding reported low numbers of patients recruited. Unlike for ARTs, access to essential NCD medications is a global challenge albeit some on-going initiatives at various international levels to address this gap [42, 43]. According to 2018 MOH’s health sector resource mapping report, the largest funding gap by cost category was for medicines and commodities, and within this category, the percentage of budget allocation by the NCD programme area was less than 2% compared to 71% for the ART programme. A recent study conducted in Zimbabwe on utilisation of health care and burden of out of pocket (OOP) expenditure showed how expenses for NCD care can result in catastrophic health expenditure [44]. Therefore funding models for affordable NCD medications and laboratory consumables need to be considered. Furthermore, forecasting and quantification of consumption of NCD medications is a challenge as the real needs are not known until access to treatment is widened. The mismatch between the demand for NCD medications and supply has been described in other LMIC [45]. Choice of medications and laboratory investigations should adapt optimal gold standards according to cost-effectiveness.

### Limitations of this study

Full support for mentorship and subsidies for medication and laboratory reagents were sustained throughout the duration of the study. We are therefore not able to ascertain the actual performance of the programme when partner-support is removed. Although we consider our intervention as successful per se, we did not assess its impact on the delivery of other PHC services, such as maternal and child health and acute emergencies. We also did not assess formally the acceptability amongst health workers or patients.

## Conclusions

In this paper we share our experience of adapting the strategies that were successful in implementing HIV programmes for DM and HTN through a 9 point conceptual framework, offering grass roots level lessons for programme managers. Overall the health system was receptive to nurse-led DM and HTN care. In particular, decentralization with a flexible integration approach for DM and HTN alongside other services is worthwhile considering. Structured mentoring of nurses on technical knowledge and practice and on organisational aspects should be considered as a key enabler to implement this model. Managers should opt for POC devices for baseline assessment, monitoring of disease progression and evaluation of treatment response. However, free medications, as with the ART programme, are currently not feasible. Instead programme managers may need to consider low cost medications affordable to the patients.

NCD-specific leadership should be considered at provincial and district level to ensure ownership and on-going mentoring support and supervision. The effectiveness of this nurse-led model needs to be further analysed.

## Supplementary information

**Additional file 1.** Zimbabwe HTN and DM Guidelines. Context-adapted simplified guidelines for management of HTN and DM in Zimbabwe developed during the study. View as.

**Additional file 2.** Indicators for DM and HTN. Indicators developed to monitor and evaluate program performance. View as.

## Data Availability

Data sharing is not applicable to this article as no datasets were analysed during the current study.

## Abbreviations

ART: Antiretroviral Therapy
DM: Diabetes Mellitus
DSD: Differentiated Service Delivery
FBS: Fasting Blood Sugar
HbA1C: Glycosylated haemoglobin
HIV: Human Immunodeficiency Virus
HTN: Hypertension
ICCC: Integrated Chronic Care Clinic
LMIC: Low- and Middle- Income Country
MOH: Ministry of Health and Child Care
MSF: Médecins Sans Frontières
NCD: Non-Communicable Diseases
NGO: Non-Governmental Organisation
OOP: Out of Pocket
OPD: Outpatients Department
PHC: Primary Health Care
RBS: Random Blood Sugar
SSA: Sub-Saharan Africa
WHO: World Health Organisation
