# Supplementary material for: Setting up a nurse-led model of care for management of hypertension and diabetes mellitus in a high HIV prevalence context in rural Zimbabwe: a descriptive study
Source: BMC Health Serv Res. 2020 Jun 1;20:486. doi: 10.1186/s12913-020-05351-x (PMC7268639; doi:10.1186/s12913-020-05351-x)
Supplement: Supplementary file 1 — Additional file 1. Zimbabwe HTN and DM Guidelines. Context-adapted simplified guidelines for management of HTN and DM in Zimbabwe developed during the study. View as. [file 12913_2020_5351_MOESM1_ESM.pdf]

GUIDELINES FOR THE MANAGEMENT OF

DIABETES

AND

HYPERTENSION

MOHCC-MSF NCD PILOT
MANICALAND PROVINCE
ZIMBABWE

Introduction

- Cardiovascular disease (CVD) is common especially above 60 years of age
- It includes coronary heart disease (e.g. myocardial infarction), cerebrovascular disease (e.g. stroke) and other manifestations
- There are many risk factors, some of which can be modified to prevent CVD. For example, early detection and management of Diabetes mellitus (DM) and Hypertension (HTN) can prevent CVD
- Some patients present with multiple risk factors and their cardiovascular risk can be assessed by using the WHO charts in annex
- The cardiovascular risk assessment will guide the prevention and treatment that we offer patients
- These are guidelines for diagnosis and management of DM and/or HTN in non pregnant adults at primary health care level

For the management of **pregnant women** with DM and or HTN, please refer to the doctor, and manage according to BEMNOC (Basic emergency newborn and obstetric care) guidelines

CHAPTER 1

MANAGEMENT OF NON PREGNANT ADULTS WITH TYPE 2 DIABETES MELLITUS

Content

- Facts about DM
- Classification of Diabetes Mellitus (DM)
- Reference ranges of blood glucose and glycated hemoglobin
- Glycemic targets or treatment goals in T2DM management
- Screening and diagnosis of symptomatic patients
- Screening and diagnosis of asymptomatic patients: when A1c is unavailable
- Screening and diagnosis of asymptomatic patients: when A1c is available
- At diagnosis: Minimum clinical and laboratory work up of patients with DM
- Management of T2DM
    - Management of T2DM: A1c not available
    - Management of T2DM: A1c 6.5 to 7.9%
    - Management of T2DM: A1c 8 to 9.9%
- Hypertension in diabetic patients
- Management of hypoglycemia at home

Facts about Diabetes Mellitus

- Diabetes mellitus is a metabolic disorder leading to chronic hyperglycemia
- Patients frequently present with concurrent hypertension (HTN)
- Patients have a higher risk of cardiovascular diseases (CVD) such as stroke

Classification of DM

Type 1 diabetes mellitus (T1DM)

- Secondary to lack of insulin production => insulin deficiency
- Typical onset in childhood but can present at ANY AGE!
- Requires daily injections of insulin

Type 2 diabetes mellitus (T2DM)

- Secondary to insulin resistance which may be accompanied by variable degrees of insulin secretory failure in advanced disease
- Is largely the result of excess body weight, unhealthy diet, physical inactivity and genetics
- Typical onset as adult

Pre-diabetes mellitus

- high blood sugar level not yet in diabetes range but risk factor for development of overt DM later on; can be addressed with diet and lifestyle changes

Gestational diabetes (GDM)

- Occurs during the 2nd and 3rd trimesters of pregnancy
- Women are at risk of developing T2DM later in life
- Due to insulin-resistance caused by placental hormones, usually glucose metabolism returns to normal after delivery

Reference ranges of blood glucose and glycated hemoglobin

RBS (mmol/L)	FBS (mmol/L)	A1c (%)	Interpretation
3.9 – 11.0	3.9 – 6.0	< 5.7	Normal
	6.1 – 6.9	5.7 – 6.4	Pre-DM

- RBS – Random blood sugar
- FBS – Fasting blood sugar
  - Fasting = no caloric intake for at least eight hours
- A1c – Glycated hemoglobin. This test measures the average blood sugar for a period of 3 months prior to the date of test

Glycemic targets or treatment goals in type 2 diabetes management

- A1c is the gold standard to measure glycemic control
- In the absence of A1c, FBS can be used

Treatment targets:

- A1c: < 7% for most patients
  Consider A1c 7.0 to 7.9% for patients at high risk of hypoglycemia:
    - Elderly,
    - History of severe hypoglycemia
    - Multiple comorbidities
    - Long standing diabetes
    - Limited life expectancy
    - Advanced complications
- FBS: < 7 mmol/L
  Consider 7 to 8 mmol/L for high risk patients (see above)

Screening & Diagnosis of Symptomatic Patients

Symptoms of DM

- ***Acute*** *(Classic symptoms)*: polydipsia, polyuria, unplanned weight loss, polyphagia, tiredness
- ***Chronic***: Recurrent UTIs, recurrent candida infections, slow healing foot cuts or wounds, peripheral neuropathy

↓

FBS or RBS
on handheld device

↓

FBS ≥ 7.0 mmol/L or
RBS ≥ 11.1 mmol/L

↓

Refer
*DM type 1?
Advanced type 2 DM?
Do baseline A1c!

FBS < 7.0 mmol/L, or
RBS < 11.1 mmol/L

↓

No DM
Look for other causes of the symptoms

*Consider diagnosis of T1DM:

- Acute classic symptoms and or
- Age < 25 years and or
- Ketonuria and or
- BMI < 25

Screening & Diagnosis of Asymptomatic Patients

When A1c testing is unavailable

Screen high risk patients

- First degree family history of DM
- Hypertension
- Active TB
- Obesity (BMI ≥ 30 kg/m²)
- Cardiovascular disease (CVD): stroke, ischaemic heart disease, heart failure, peripheral artery disease
- Chronic Kidney Disease (CKD): CrCl < 60 ml/min

↓

FBS on handheld device in mmol/L

↓

FBS ≥ 7.0

→ Repeat **FBS** in 1 week

- FBS ≥ 7.0 → **Diabetes confirmed!** → Follow management cascade for confirmed T2DM
- FBS 6.1 to 6.9 → Impaired FBS →
  - Dietary & lifestyle counseling
  - Re-screen after 6 months
- FBS 3.9 to 6.0 → Discrepancy between 1st and 2nd test → **Refer**

FBS < 7.0*

→ **No DM** →

- Dietary & lifestyle counseling
- Re-screen after 3 years

** A normal RBS is not a good predictor of DM in asymptomatic patients*

Screening & Diagnosis of Asymptomatic patients

When A1c testing is available

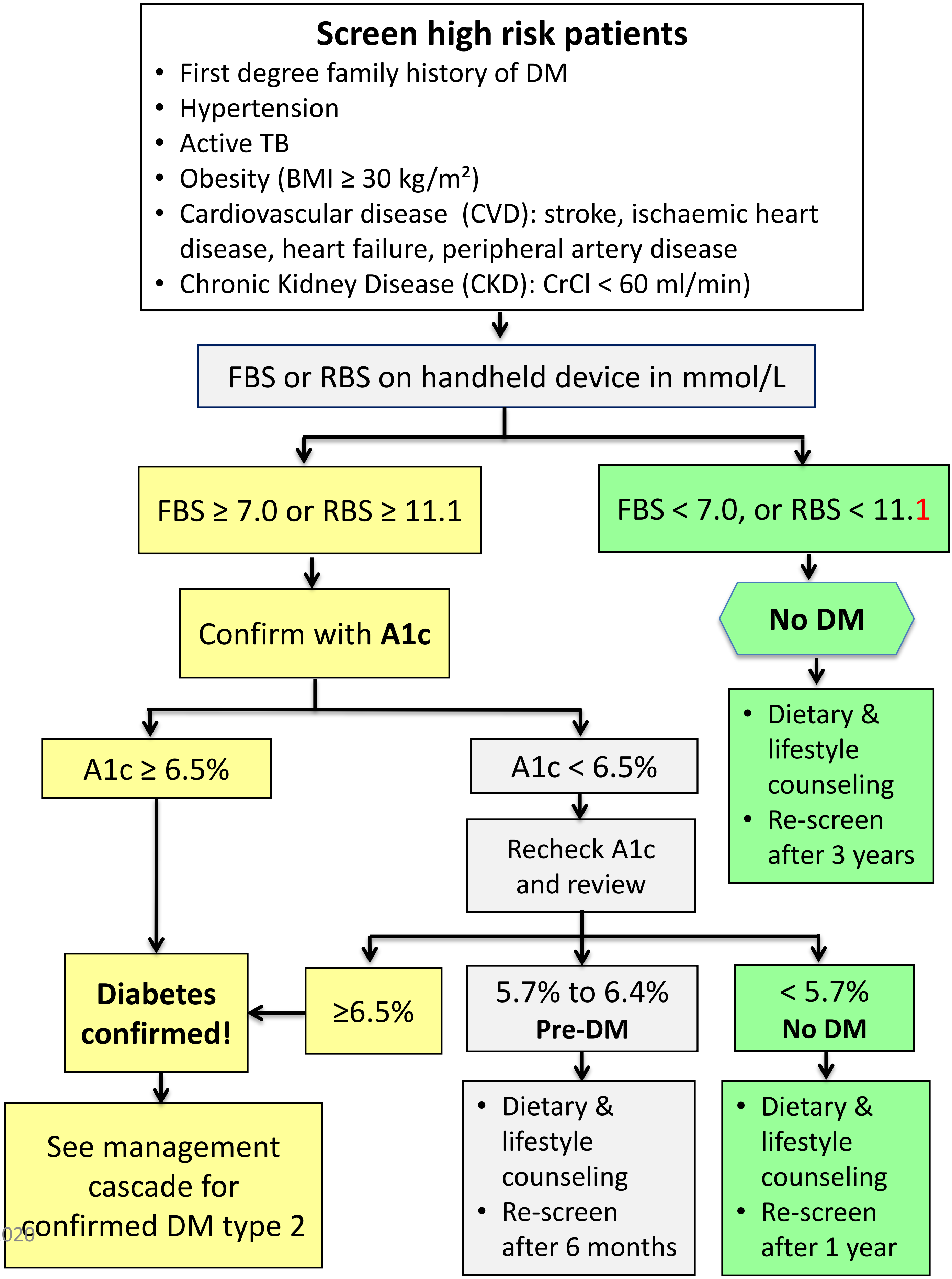

At diagnosis: minimum clinical and laboratory work up of patients with DM

- **Symptoms and signs:** shortness of breath or cough, fatigue, leg swelling (oedema), irregular pulse or rapid heart rate, pallor
- Weight, height, BMI
- Urinalysis – document presence or absence of proteinuria
- Serum creatinine and creatinine clearance
- Fasting blood sugar
- Total cholesterol if available
- Assess cardiovascular risk

Management of T2DM

Key notes

- *First line* oral hypoglycemic agents (OHA) are biguanides e.g. metformin

- *Second line* OHA are sulphonylureas:
  - Gliclazide and Glimepiride should be **preferred** where resources permit
  - Glibenclamide should not be used ≥ 60 years of age and if creatinine clearance < 60 ml/min !

- All patients presenting with *A1c of 10%* or more should be considered for insulin initiation

All serious adverse events must be reported to the MEDICAL RESEARCH COUNCIL OF ZIMBABWE and MEDICINES CONTROL AUTHORITY OF ZIMBABWE using the *SERIOUS ADVERSE EVENT REPORTING FORM*

MANAGEMENT OF T2DM

When A1c testing is not available

- **Define treatment goal: FBS < 7 mmol/L**
- **Take sample for CrCL at day 1 of treatment initiation**
- **Counsel on diet & lifestyle**

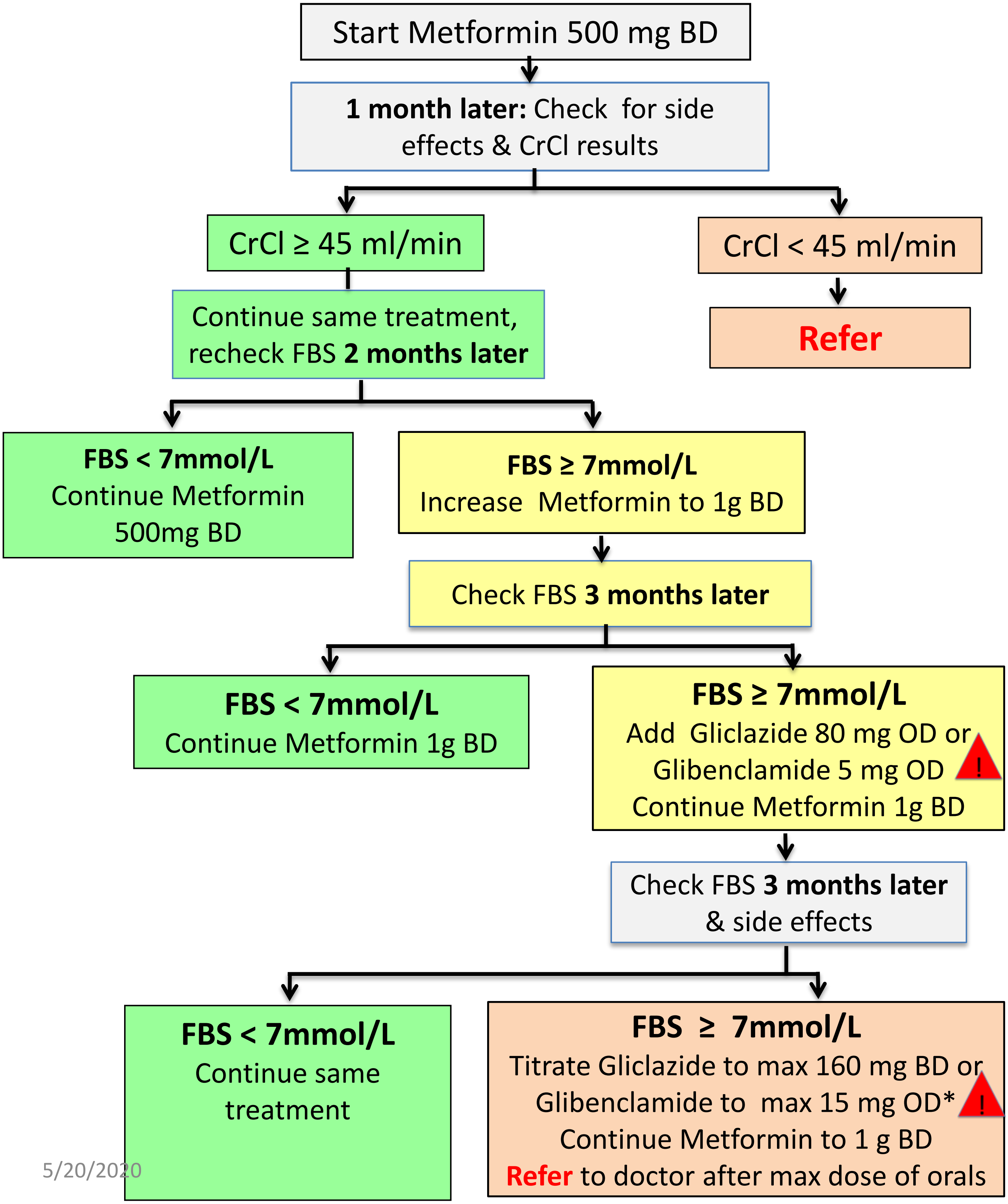

MANAGEMENT OF DIABETES TYPE 2

When A1c is 6.5 to 7.9%

- **Define treatment goal (CAUTION FOR HIGH RISK PATIENTS!)**
- **Take sample for CrCL at treatment initiation**
- **Counsel on diet & lifestyle**
- **Check treatment/diet compliance & side effects each visit**

Start Metformin 500 mg BD

↓

1 month later:
Check CrCl result

↓

CrCl ≥ 45 ml/min → Continue same treatment, recheck A1c **2 months later**

CrCl < 45 ml/min → **Refer**

↓ (after 2 months)

A1c < 7%
Continue Metformin 500 mg BD

A1c 7 to 7.9%
Increase Metformin to 1g BD

A1c ≥ 8%
Move on to dual therapy. See flowchart A1c ≥ 8%

MANAGEMENT OF T2DM

When A1c is 8 to 9.9%

- **Define treatment goal; (CAUTION FOR HIGH RISK PATIENTS!)**
- **Take sample for CrCL at treatment initiation. Counsel on diet & lifestyle**

Start dual therapy: Metformin 500 mg BD
AND Gliclazide 80 mg OD or Glibenclamide 5 mg OD !

↓

1 month later:
Check clinical tolerance & CrCl

- CrCl ≥ 45 ml/min → Continue same treatment → Check A1c **2 months later**
  - **A1c < 7%** — Continue same treatment
  - **A1c ≥ 7%** — Increase Metformin to 1 g BD. Continue Gliclazide or Glibenclamide as above !
    ↓
    Check A1c **3 months later**
    - **A1c < 7%** — Continue same treatment
    - **A1c ≥ 7%** — Increase Gliclazide to 80 mg BD or Glibenclamide to 10 mg OD. Continue Metformin 1g BD !
      ↓
      Check A1c **3 months later**
      - **A1c < 7%** — Continue last prescribed treatment
      - **A1c ≥ 7%** — Titrate Gliclazide to max 160 mg BD or Glibenclamide to max 15 mg OD. Continue Metformin to 1 g BD. **Refer** to doctor after max dose of orals !
- CrCl < 45 ml/min → **Refer**

Hypertension in diabetic patients

- Hypertension is a common problem in patients with diabetes
- The diabetes-hypertension co-morbidity increases the risk for cardiovascular morbidity and mortality
- Therefore all patients with DM and HTN must be put on treatment whatever is the stage of HTN
- See HTN diagnostic and treatment cascades (page 19 to 28)

Management of hypoglycemia at home

Step 1: First, eat or drink 10 to 15 grams of a fast-acting carbohydrate, such as:

- Four to six pieces of hard sweets (not sugar-free)
- ½ (125ml) cup of fruit juice
- 1 cup of milk
- ½ (125ml) cup of soft-drink (not sugar-free)
- 1 tablespoon honey (put it under your tongue so it gets absorbed into your bloodstream faster)

Step 2:

- Wait 15 minutes, re-check your blood glucose levels to see if your blood glucose level has risen above 3.9 mmol/L.
- If your blood glucose level has risen above 3.9 mmol/L go to Step 3.
- If your blood glucose level is still below 3.9 mmol/L, repeat Step 1.

Step 3: Eat a snack or meal with longer acting carbohydrate. Chose one among the following:

- A slice of bread with peanut butter or avocado
- A small sweet potato
- A fruit: banana, orange, apple, mango…
- A handfull of peanuts, mutakura, nyimo
- 2-3 pieces of dried fruit
- 1 cup (125 ml) of natural yoghurt or mukaka wakakora
- A fist of sadza, pasta, rice

CHAPTER 2

MANAGEMENT OF HYPERTENSION (HTN)

Content

- Blood pressure measurement – SOP
- Frequency of BP monitoring and practical considerations in checking BP
- Treatment goals for HTN
- HTN diagnostic cascade
- At diagnosis: Minimum clinical and laboratory work-up of patients with HTN
- HTN treatment cascades:
  - Stage 1 HTN: patients with HTN-only
  - Stage 1 HTN: patients with DM or high CVD risk
  - Stage 2 HTN
  - Stage 3 HTN

BP Measurement
Standard Operating Procedure

Factor	Procedure	Explanation
**General**	First consultation, take reading from the left arm of patient<br>For diagnosis of HTN, take readings on 3 different visits over 2 weeks to 2 months. If ≥2 out of 3 of the BP readings are >140/90 then patient has HTN<br>For BP > 180/110 (stage 3 HTN), let the patient rest one hour, repeat and if persistently > 180/110 refer to severe HTN management protocol	▪ BP varies throughout day.<br>▪ Be consistent, always take the BP from the same arm
**Type of blood pressure machine**	Use an automated BP machine with correctly fitting cuff	▪ Manual devices subject to human error. Small cuffs give high readings.
**What the patient /client should do**	Stop talking during the procedure and be relaxed<br>Sit with back supported. Avoid crossed legged position.<br>Client's arm at level of heart, arm resting on table.<br>Avoid distended bladder (Discomfort and pain raise BP. A full bladder can be painful).	▪ Unsupported back raises BP by 6mmHg, talking by 8 to 15mmHg<br>▪ Crossed legs raises BP by 2 to 8 mmHg.<br>▪ Hanging arms raise BP by 10 to 12mmHg.
**What the health care provider should do**	Remove restrictive clothing from arm. Instruct client to relax arm in use<br>Place BP cuff with bladder midline over brachial artery.<br>Stop talking during BP measurement.<br>Press Start button for the reading to be taken.<br>If BP >140/90, take at least 2 BP readings and record the lowest value<br>Note the circumstances under which BP is measured:<br>▪ fever, presenting complaint, stress<br>▪ If patient is on antihypertensive medications, record 2 previous documented BP values, date and circumstances	▪ Stress, caffeine, antihypertensive medications and physical activity affect blood pressure

Treatment goals for hypertension

- Aim for sustained long-term BP control
- Avoid aggressive treatment.
- Acute changes in BP put the patient at risk of complications
- **Treatment goal for all patients: < 140/90 mmHg**

HTN Diagnostic Cascade

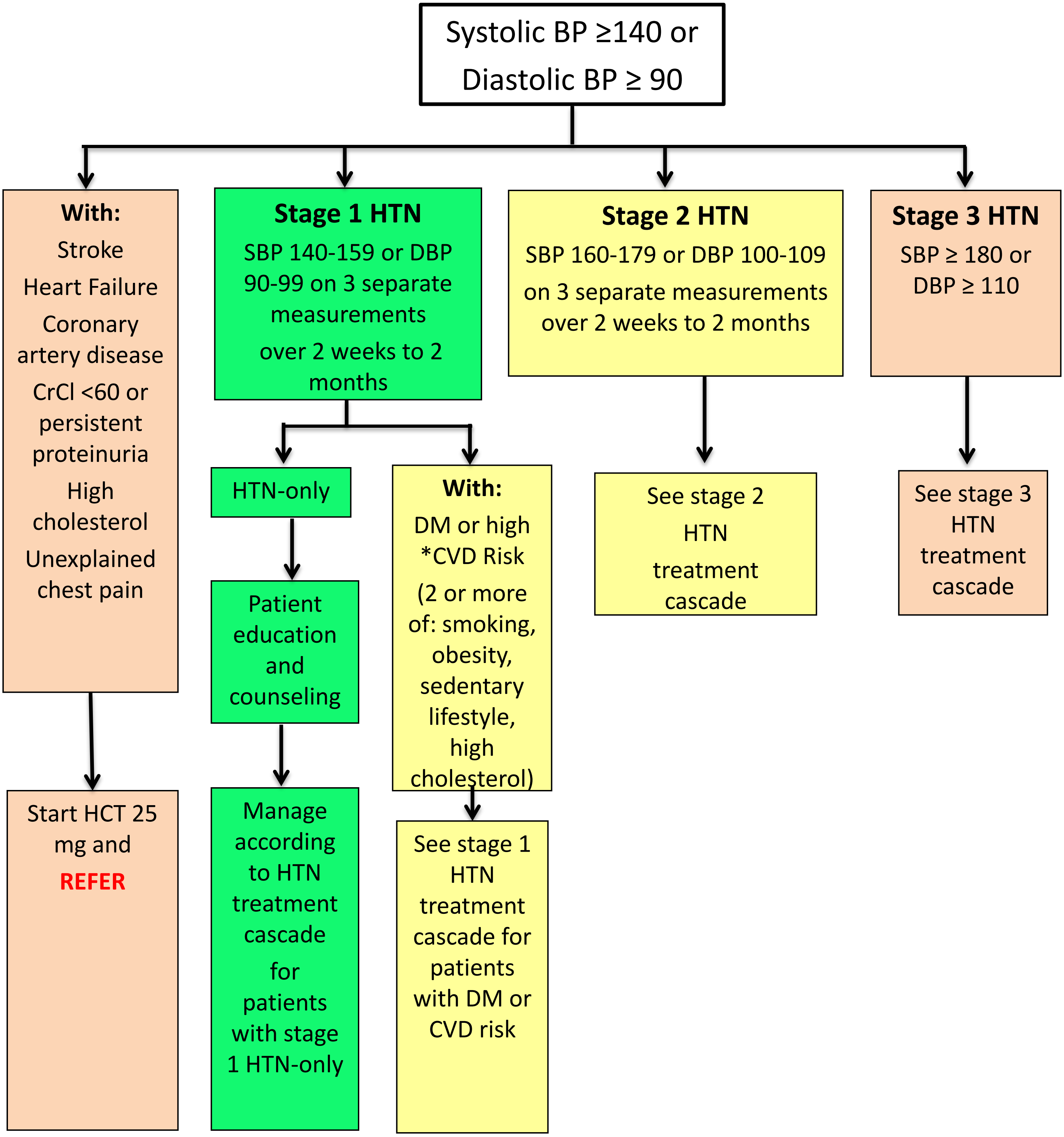

At diagnosis: minimum clinical and laboratory work up of patients with HTN

- **Symptoms and signs:** shortness of breath or cough, fatigue, leg swelling (oedema), irregular pulse or rapid heart rate, pallor
- Weight, height, BMI
- Urinalysis – document presence or absence of proteinuria
- Serum creatinine and creatinine clearance
- Fasting blood sugar
- Total cholesterol if available
- Assess cardiovascular risk

STAGE 1 HTN TREATMENT CASCADE
Patients with HTN-only
Preferred practice if resources permit

BP ≥ 140/90 mmHg and <160/100mmHg
- No diabetes mellitus
- *No cor-mobidities*
- Program Target: BP < 140/90 for ALL PATIENTS

↓

- Refer to counselor for educational session and prescribe lifestyle management:
- Low salt diet
- Regular exercises (minimum 30 min/day for 5-7 days per week of moderate intensity exercises)
- Stop smoking
- Aim for weight reduction to chive BMI < 25
- **REVIEW AFTER 6 MONTHS**

↓

If BP still above target: START HCT 25 mg OD
Reinforce lifestyle changes
Review in 4 weeks with CrCl

↓

CrCl ≥ 45 ml/min

↓

BP still above target despite good adherence: add Amlodipine 5 mg daily and manage as Stage 2 hypertension

CrCl < 45 ml/min

↓

Refer to Secondary Level
If BP still above target:
- Consider withholding HCT. ***Reduced efficacy of HCT***
- Start Amlodipine 5 mg daily

STAGE 1 HTN TREATMENT CASCADE
PATIENTS WITH DM OR HIGH CVD RISK

BP ≥ 140/90 mmHg AND < 160/100
- Take sample for creatinine clearance (CrCl) and start treatment
- *Ideal target BP for DM-HTN: <130/80mmHg*
- **Program Target:** BP < 140/90 mmHg

↓

START HCT 25 mg OD.
Review in 4 weeks with CrCl

↓

CrCl ≥ 45 ml/min

↓

If BP still above target: confirm adherence and add **AMLODIPINE 5 mg OD.** Review in 4 weeks

↓

If BP still above target : confirm adherence and increase **AMLODIPINE** to 10 mg OD. Review in 4 weeks

↓

Refer

↓

If BP still above target: confirm adherence and **add ENALAPRIL 10 mg OD.** Review in 4 weeks

If BP still above target: confirm adherence and **increase ENALAPRIL to 20 mg OD.** Review in 4 weeks

If BP still above target: confirm adherence and **increase ENALAPRIL to 40 mg OD** Review in 4 weeks

If BP still above target , consider **start of ATENOLOL)**

CrCl < 45 ml/min

↓

Refer

↓

Consider withholding HCT. ***Reduced efficacy of HCT***

Start AMOLDIPINE 5 mg OD. Review in 4 weeks

If BP still above target : confirm adherence and **increase AMLODIPINE to 10 mg OD.** Review in 4 weeks

If BP still above target: Consider adding enalapril, atenolol or spironolactone depending on CrCl and provider experience

SECONDARY CARE LEVEL

STAGE 2 HTN TREATMENT CASCADE

BP ≥ 160-179/100 -109 mmHg AND < 180/110

- Sample for creatinine clearance (CrCl)
- Start treatment
- Target: BP < 140/90 FOR ALL PATIENTS

↓

START HCT 25 mg OD AND Amlodipine 5 mg OD
Review in 4 weeks with CrCl

CrCl ≥ 45 ml/min

↓

If BP still above target despite adherence: increase AMLODIPINE to 10 mg OD. Review in 4weeks

↓

If BP still above target despite adherence: **Refer**

SECONDARY CARE LEVEL

If BP still above target: confirm adherence and add ENALAPRIL 10 mg OD. Review in 4 weeks

If BP still above target: confirm adherence and increase ENALAPRIL to 20 mg OD or 20 mg OD. Review in 4 weeks

If BP still above target: confirm adherence and increase ENALAPRIL to 40 mg OD Review in 4 weeks

If BP still above target : confirm adherence and consider start of SPIRONOLACTONE or ATENOLOL

CrCl < 45 ml/min

↓

Refer to secondary level

If BP still above target: Increase AMOLDIPINE to 10 mg OD, Review in 4 weeks

If BP still above target: Consider adding enalapril, atenolol or spironolactone depending on CrCl and provider experience

STAGE 3 HTN TREATMENT CASCADE

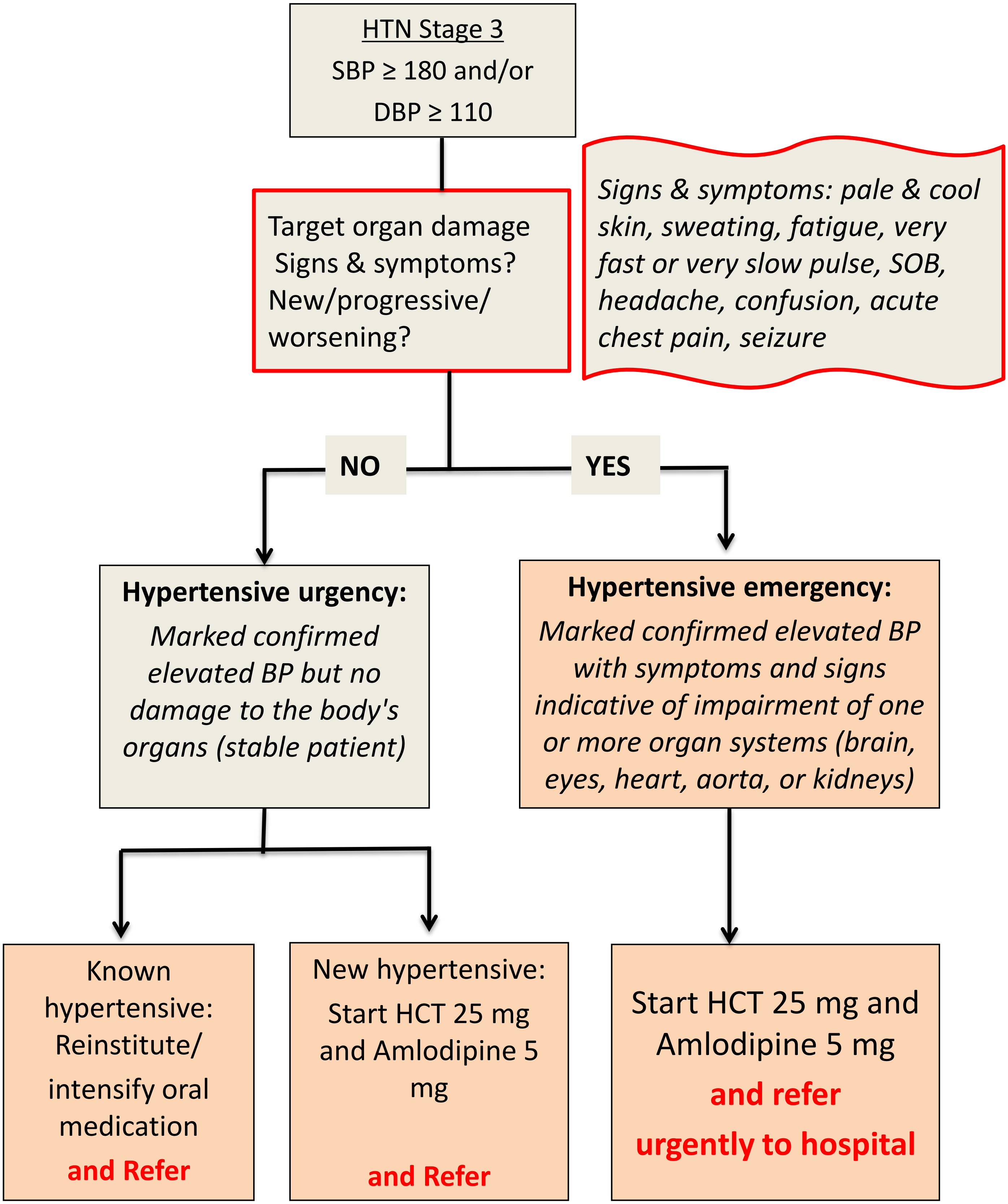

Review patients after 3 days. If follow up BP < 180/110 titrate medication upwards and encourage review with the doctor. If persistently high, emphasize need for referral!

Appendix
I - IX

Appendix I
T2DM: Follow up schedule for examinations and laboratory tests

	Baseline	3 monthly	6 monthly	Yearly
**Symptom screen including TB**	X	Every clinical visit		
**HbA1c**	X	Until at goal	X	
**BP**	X	Every clinical visit		
**Weight and BMI**	X		X	
**Fasting blood glucose**	X	Where no A1c testing		
**Foot exam**	X	Every clinical visit		
**Creatinine Clearance**	X	If baseline CrCl < 60 ml/min		**X**
**Doctor consultation**				**X**

**Appendix II<br>HTN: Follow up schedule for examinations and lab tests**				
	**Baseline**	**3 monthly**	**6 monthly**	**Yearly**
**Symptom screen including TB**	X	Every clinical visit		
**BP**	X	Every clinical visit		
**Weight and BMI**	X		X	
**Screening for DM with questionnaire**	X	If positive symptom screen, do FBS	If positive symptom screen, do FBS	**If positive symptom screen, do FBS**
**Fasting blood glucose**	X			**X**
**Urinalysis**	X	If baseline abnormal		**X**
**Creatinine Clearance**	X	If baseline CrCl < 60 ml/min		**X**
**Doctor's consultation**				**x**

Appendix III: Staging of kidney function

Stage	Cr Cl	Description	Treatment
1	>90	Normal function	Observation, control of BP
2	60−89	Mildly reduced	Observation, control of BP and risk factors
3	30−59	Moderately reduced	Observation, control of BP and risk factors
4	15−29	Severely reduced;	Planning for end-stage renal failure
5	<15	End stage	Dialysis referral if resources available

Chronic Kidney Disease (CKD) = CrCl <60ml/min measured on at least two occasions ≥3months apart

Appendix IV: Medication adjustment according to kidney function (Creatinine clearance in ml/min)

	CrCl ≥ 60	CrCl 60-30	CrCl < 30	CrCl < 15
**Enalapril**	10mg to 40mg	5mg to 40mg	2.5mg to 40mg	Avoid
**Atenolol**	25mg to 100mg OD		Reduce dose, max of 50mg/day	Reduce dose, max of 25mg/day
**Furosemide**	20mg to 80mg BD		No dose adaptation	
**Amlodipine**	5mg to 10mg OD		No dose adaptation	
**HCT**	25mg OD		Reduced efficacy. Consider alternative	Avoid at CrCl <10
**Spironolactone**	12.5mg to 50mg OD		Avoid	Avoid
**Metformin**	500mg to 1g BD	ClCr <45, avoid initiation, if already on metformin, max dose should be ≤ 1g OD	Avoid	
**Glibenclamide**	5mg to 10mg OD		Avoid	
**Gliclazide**	80mg OD to 320mg, split into BD if ≥ 160 mg		Avoid	
**Atorvastatin**	10mg to 80mg<br>Max dose with Protease Inhibitors = 20mg OD			

Appendix V
Criteria for prescribing Aspirin for the prevention of CVD

Dose of aspirin: 75 to 150 mg (depending on availability)

Risk category	Beneficiaries
**Secondary prevention** *(Previous CVD event such as MI, heart failure, stroke, peripheral vascular disease, unexplained chest pain, atrial fibrillation)*	Any patient with previous CVD event (independently of age)

Note: Aspirin should not be used in the routine primary prevention of atherosclerotic CVD due to lack of net benefit *(ref. AHA 2019)*

Appendix VI
Criteria for prescribing Statins for the prevention of CVD

Note: Statins to be prescribed at district level, refills at primary health care level

Risk category	Beneficiaries	Dose of Atorvastatin
**Primary prevention** *(To prevent the occurrence of a cardio-vascular event in people who have not yet had one)*	HIgh Risk patients only: DM > 40 yrs old	40 mg daily
**Secondary prevention** *(Previous CVD event such as MI, heart failure, stroke, atrial fibrillation, peripheral vascular disease)*	Any patient with previous CVD event	40 to 80 mg daily If on Protease Inhibitor, 10-20mg daily

Appendix VII

WHO cardiovascular disease risk laboratory based charts

Southern Sub-Saharan Africa: People without DM

Botswana, Lesotho, Namibia, Swaziland, South Africa, Zimbabwe.

Risk Level: <5% | 5% to <10% | 10% to <20% | 20% to <30% | ≥30%

People without Diabetes

Age (years)	SBP (mmHg)	Men Non-smoker <4	4-4.9	5-5.9	6-6.9	≥7	Men Smoker <4	4-4.9	5-5.9	6-6.9	≥7	Women Non-smoker <4	4-4.9	5-5.9	6-6.9	≥7	Women Smoker <4	4-4.9	5-5.9	6-6.9	≥7
70-74	≥180	21	23	26	28	31	27	29	32	35	39	18	19	19	20	21	25	26	27	28	29
70-74	160-179	18	19	21	23	26	22	24	27	30	33	15	16	17	17	18	21	22	23	24	25
70-74	140-159	14	16	17	19	21	18	20	22	25	27	13	13	14	15	15	18	19	19	20	21
70-74	120-139	12	13	14	16	18	15	17	18	20	23	11	11	12	12	13	15	16	16	17	18
70-74	<120	10	11	12	13	14	12	14	15	17	19	9	10	10	10	11	13	13	14	15	15
65-69	≥180	17	18	20	22	25	22	25	27	30	34	14	15	15	16	17	21	22	23	24	25
65-69	160-179	13	15	16	18	20	18	20	22	25	28	12	12	13	13	14	17	18	19	20	21
65-69	140-159	11	12	13	15	16	14	16	18	20	23	10	10	11	11	12	14	15	16	17	18
65-69	120-139	8	9	10	12	13	12	13	14	16	18	8	8	9	9	10	12	13	13	14	15
65-69	<120	7	7	8	9	11	9	10	12	13	15	6	7	7	8	8	10	10	11	12	12
60-64	≥180	13	14	16	18	20	18	21	23	26	29	11	11	12	13	14	17	18	19	21	22
60-64	160-179	10	11	12	14	16	15	16	18	21	23	9	9	10	10	11	14	15	16	17	18
60-64	140-159	8	9	10	11	13	11	13	14	16	19	7	7	8	8	9	11	12	13	14	15
60-64	120-139	6	7	8	9	10	9	10	11	13	15	6	6	6	7	7	9	10	11	11	12
60-64	<120	5	5	6	7	8	7	8	9	10	12	5	5	5	6	6	8	8	9	9	10
55-59	≥180	10	11	12	14	16	15	17	19	22	25	8	9	9	10	11	14	15	16	18	19
55-59	160-179	8	8	10	11	13	12	13	15	17	20	6	7	7	8	9	11	12	13	14	15
55-59	140-159	6	6	7	8	10	9	10	12	13	16	5	5	6	6	7	9	10	11	11	12
55-59	120-139	4	5	6	6	7	7	8	9	10	12	4	4	5	5	5	7	8	8	9	10
55-59	<120	3	4	4	5	6	5	6	7	8	9	3	3	4	4	4	6	6	7	7	8
50-54	≥180	8	9	10	11	13	12	14	16	19	22	6	7	7	8	9	12	13	14	15	17
50-54	160-179	6	6	7	8	10	9	11	12	14	17	5	5	6	6	7	9	10	11	12	13
50-54	140-159	4	5	5	6	7	7	8	9	11	13	4	4	4	5	5	7	8	9	9	10
50-54	120-139	3	3	4	5	6	5	6	7	8	10	3	3	3	4	4	6	6	7	7	8
50-54	<120	2	3	3	4	4	4	4	5	6	7	2	2	3	3	3	4	5	5	6	6
45-49	≥180	6	7	7	9	10	10	12	13	16	19	5	5	6	6	7	10	11	12	13	14
45-49	160-179	4	5	5	6	8	7	9	10	12	14	4	4	4	5	5	8	8	9	10	11
45-49	140-159	3	3	4	5	6	5	6	7	9	10	3	3	3	4	4	6	6	7	8	9
45-49	120-139	2	3	3	3	4	4	5	5	6	8	2	2	2	3	3	4	5	5	6	7
45-49	<120	2	2	2	3	3	3	3	4	5	6	2	2	2	2	2	3	4	4	5	5
40-44	≥180	5	5	6	7	8	8	10	11	13	16	4	4	4	5	5	8	9	10	11	12
40-44	160-179	3	4	4	5	6	6	7	8	10	12	3	3	3	4	4	6	7	7	8	9
40-44	140-159	2	3	3	3	4	4	5	6	7	9	2	2	2	3	3	5	5	6	6	7
40-44	120-139	2	2	2	3	3	3	4	4	5	6	1	2	2	2	2	3	4	4	5	5
40-44	<120	1	1	2	2	2	2	3	3	4	5	1	1	1	2	2	3	3	3	4	4

Total cholesterol (mmol/l)

Appendix VIII
WHO cardiovascular disease risk laboratory based charts
Southern Sub-Saharan Africa: People with DM
Botswana, Lesotho, Namibia, Swaziland, South Africa, Zimbabwe.

Risk Level: <5% | 5% to <10% | 10% to <20% | 20% to <30% | ≥30%

People with Diabetes

Age (years)	SBP (mmHg)	Men										Women									
		Non-smoker					Smoker					Non-smoker					Smoker				
	Total cholesterol (mmol/l)	<4	4-4.9	5-5.9	6-6.9	≥7	<4	4-4.9	5-5.9	6-6.9	≥7	<4	4-4.9	5-5.9	6-6.9	≥7	<4	4-4.9	5-5.9	6-6.9	≥7
70-74	≥180	29	31	35	38	42	36	39	43	47	51	26	27	28	30	31	35	37	38	40	41
70-74	160-179	24	26	29	32	35	30	33	36	40	44	23	23	24	25	27	31	32	33	34	36
70-74	140-159	20	22	24	27	29	25	28	30	34	37	19	20	21	22	23	26	27	28	30	31
70-74	120-139	16	18	20	22	25	21	23	25	28	31	16	17	18	18	19	22	23	24	25	27
70-74	<120	13	15	16	18	20	17	19	21	23	26	14	14	15	16	16	19	20	21	22	23
65-69	≥180	24	26	29	32	36	32	35	39	43	47	22	23	24	25	27	32	33	35	37	39
65-69	160-179	19	21	24	27	30	26	29	32	36	40	18	19	20	21	22	27	28	30	31	33
65-69	140-159	16	17	19	22	24	21	24	26	30	33	15	16	17	18	19	23	24	25	26	28
65-69	120-139	12	14	16	18	20	17	19	21	24	27	13	13	14	15	16	19	20	21	22	24
65-69	<120	10	11	13	14	16	14	15	17	20	22	10	11	12	12	13	16	17	18	19	20
60-64	≥180	20	22	25	28	31	28	31	35	39	44	18	19	20	22	23	28	30	32	34	36
60-64	160-179	16	17	20	22	25	22	25	28	32	36	15	16	17	18	19	24	25	27	28	30
60-64	140-159	12	14	16	18	20	18	20	23	26	29	12	13	14	15	16	19	21	22	24	25
60-64	120-139	10	11	12	14	16	14	16	18	21	24	10	10	11	12	13	16	17	18	19	21
60-64	<120	7	8	10	11	13	11	13	14	17	19	8	8	9	10	10	13	14	15	16	17
55-59	≥180	16	18	21	23	27	25	28	31	36	40	15	16	17	18	20	25	27	29	31	34
55-59	160-179	13	14	16	18	21	19	22	25	28	33	12	13	14	15	16	21	22	24	26	28
55-59	140-159	10	11	12	14	17	15	17	20	23	26	9	10	11	12	13	17	18	19	21	23
55-59	120-139	7	8	10	11	13	12	13	15	18	21	7	8	9	9	10	13	14	16	17	18
55-59	<120	6	6	7	9	10	9	10	12	14	16	6	6	7	8	8	11	12	13	14	15
50-54	≥180	14	15	17	20	23	22	25	28	32	37	12	13	14	15	17	23	24	26	29	31
50-54	160-179	10	11	13	15	18	17	19	22	25	29	10	10	11	12	13	18	19	21	23	25
50-54	140-159	8	9	10	12	14	13	14	17	20	23	7	8	9	10	10	14	15	17	19	20
50-54	120-139	6	6	7	9	10	10	11	13	15	18	6	6	7	7	8	11	12	13	15	16
50-54	<120	4	5	6	7	8	7	8	10	12	14	4	5	5	6	7	9	10	11	12	13
45-49	≥180	11	13	14	17	20	19	22	25	29	34	10	11	12	13	14	20	22	24	26	29
45-49	160-179	8	9	11	12	15	14	16	19	22	27	8	8	9	10	11	16	17	19	21	23
45-49	140-159	6	7	8	9	11	11	12	14	17	20	6	6	7	8	9	12	13	15	16	18
45-49	120-139	4	5	6	7	8	8	9	11	13	15	4	5	5	6	7	9	10	12	13	14
45-49	<120	3	4	4	5	6	6	7	8	10	12	3	4	4	5	5	7	8	9	10	11
40-44	≥180	9	11	12	14	17	17	20	23	26	31	8	9	10	11	12	18	20	22	24	27
40-44	160-179	7	8	9	10	12	12	14	17	20	24	6	7	7	8	9	14	15	17	19	21
40-44	140-159	5	5	6	7	9	9	10	12	15	18	5	5	6	6	7	10	11	13	15	16
40-44	120-139	3	4	5	5	7	6	8	9	11	13	3	4	4	5	5	8	9	10	11	13
40-44	<120	2	3	3	4	5	5	5	7	8	10	2	3	3	4	4	6	7	8	9	10

Total cholesterol (mmol/l)

Appendix IX:
WHO cardiovascular disease risk non-laboratory based charts
Southern Sub-Saharan Africa
Botswana, Lesotho, Namibia, Swaziland, South Africa, Zimbabwe.

Risk Level: <5% | 5% to <10% | 10% to <20% | 20% to <30% | ≥30%

Non-laboratory based risk chart

Age (years)	SBP (mmHg)	Men Non-smoker <20	20-24	25-29	30-35	≥35	Men Smoker <20	20-24	25-29	30-35	≥35	Women Non-smoker <20	20-24	25-29	30-35	≥35	Women Smoker <20	20-24	25-29	30-35	≥35
70-74	≥180	23	24	26	28	30	29	31	33	35	38	20	20	21	22	22	27	28	29	30	31
	160-179	19	20	21	23	25	24	26	28	29	31	17	17	18	18	19	23	24	25	26	26
	140-159	15	16	18	19	20	20	21	23	24	26	14	14	15	15	16	20	20	21	22	22
	120-139	12	13	14	15	17	16	17	19	20	21	12	12	12	13	13	16	17	18	18	19
	<120	10	11	12	12	13	13	14	15	16	18	10	10	10	11	11	14	14	15	15	16
65-69	≥180	18	19	21	23	25	24	26	29	31	34	15	16	17	17	18	24	24	25	26	27
	160-179	14	15	17	18	20	20	21	23	25	27	13	13	14	14	15	19	20	21	22	23
	140-159	11	12	13	14	16	16	17	19	20	22	10	11	11	11	12	16	17	17	18	19
	120-139	9	10	11	12	13	12	14	15	16	18	8	9	9	9	10	13	14	14	15	15
	<120	7	8	8	9	10	10	11	12	13	14	7	7	7	8	8	11	11	11	12	12
60-64	≥180	14	15	16	18	20	20	22	24	27	30	12	13	13	14	14	20	21	22	23	24
	160-179	11	12	13	14	16	16	17	19	21	24	10	10	10	11	11	16	17	18	18	19
	140-159	8	9	10	11	12	12	14	15	17	19	8	8	8	9	9	13	13	14	15	15
	120-139	6	7	8	9	10	10	11	12	13	15	6	6	7	7	7	10	11	11	12	12
	<120	5	5	6	7	7	7	8	9	10	12	5	5	5	5	6	8	9	9	9	10
55-59	≥180	10	12	13	15	16	17	19	21	23	26	9	10	10	11	11	17	18	19	20	21
	160-179	8	9	10	11	13	13	14	16	18	21	7	8	8	8	9	13	14	15	15	16
	140-159	6	7	7	8	10	10	11	12	14	16	6	6	6	6	7	10	11	11	12	13
	120-139	4	5	6	6	7	7	8	9	11	12	4	5	5	5	5	8	8	9	9	10
	<120	3	4	4	5	5	5	6	7	8	9	3	3	4	4	4	6	7	7	7	8
50-54	≥180	8	9	10	12	13	14	16	18	20	23	7	8	8	8	9	14	15	16	17	18
	160-179	6	7	8	9	10	10	12	13	15	18	6	6	6	6	7	11	12	12	13	14
	140-159	4	5	6	6	7	7	9	10	12	13	4	4	5	5	5	8	9	9	10	11
	120-139	3	4	4	5	5	6	6	7	9	10	3	3	3	4	4	6	7	7	8	8
	<120	2	3	3	3	4	4	5	5	6	7	2	2	3	3	3	5	5	5	6	6
45-49	≥180	6	7	8	9	11	11	13	15	18	21	6	6	6	7	7	12	13	14	15	15
	160-179	4	5	6	7	8	8	9	11	13	15	4	4	5	5	5	9	10	10	11	12
	140-159	3	4	4	5	6	6	7	8	9	11	3	3	3	4	4	7	7	8	8	9
	120-139	2	3	3	3	4	4	5	6	7	8	2	2	2	3	3	5	5	6	6	7
	<120	2	2	2	3	3	3	4	4	5	6	2	2	2	2	2	4	4	4	5	5
40-44	≥180	5	5	6	7	9	9	11	13	15	18	4	5	5	5	6	10	11	12	12	13
	160-179	3	4	4	5	6	6	8	9	11	13	3	3	4	4	4	7	8	9	9	10
	140-159	2	3	3	4	4	5	5	6	8	9	2	2	2	3	3	5	6	6	7	7
	120-139	2	2	2	3	3	3	4	5	6	7	2	2	2	2	2	4	4	4	5	5
	<120	1	1	2	2	2	2	3	3	4	5	1	1	1	1	1	3	3	3	4	4

Body mass index (kg/m^2)

Appendix X

Body Mass Index (BMI) Chart for Adults

Obese (>30) | Overweight (25-30) | Normal (18.5-25) | Underweight (<18.5)

HEIGHT in feet/inches and centimeters

**WEIGHT**		4'8"	4'9"	4'10"	4'11"	5'0"	5'1"	5'2"	5'3"	5'4"	5'5"	5'6"	5'7"	5'8"	5'9"	5'10"	5'11"	6'0"	6'1"	6'2"	6'3"	6'4"	6'5"
lbs	(kg)	142cm		147	150	152	155	157	160	163	165	168	170	173	175	178	180	183	185	188	191	193	196
**260**	**(117.9)**	**58**	**56**	**54**	**53**	**51**	**49**	**48**	**46**	**45**	**43**	**42**	**41**	**40**	**38**	**37**	**36**	**35**	**34**	**33**	**32**	**32**	**31**
255	(115.7)	57	55	53	51	50	48	47	45	44	42	41	40	39	38	37	36	35	34	33	32	31	30
**250**	**(113.4)**	**56**	**54**	**52**	**50**	**49**	**47**	**46**	**44**	**43**	**42**	**40**	**39**	**38**	**37**	**36**	**35**	**34**	**33**	**32**	**31**	**30**	**30**
245	(111.1)	55	53	51	49	48	46	45	43	42	41	40	38	37	36	35	34	33	32	31	31	30	29
**240**	**(108.9)**	**54**	**52**	**50**	**48**	**47**	**45**	**44**	**43**	**41**	**40**	**39**	**38**	**36**	**35**	**34**	**33**	**33**	**32**	**31**	**30**	**29**	**28**
235	(106.6)	53	51	49	47	46	44	43	42	40	39	38	37	36	35	34	33	32	31	30	29	29	28
**230**	**(104.3)**	**52**	**50**	**48**	**46**	**45**	**43**	**42**	**41**	**39**	**38**	**37**	**36**	**35**	**34**	**33**	**32**	**31**	**30**	**30**	**29**	**28**	**27**
225	(102.1)	50	49	47	45	44	43	41	40	39	37	36	35	34	33	32	31	31	30	29	28	27	27
**220**	**(99.8)**	**49**	**48**	**46**	**44**	**43**	**42**	**40**	**39**	**38**	**37**	**36**	**34**	**33**	**32**	**32**	**31**	**30**	**29**	**28**	**27**	**27**	**26**
215	(97.5)	48	47	45	43	42	41	39	38	37	36	35	34	33	32	31	30	29	28	28	27	26	25
**210**	**(95.3)**	**47**	**45**	**44**	**42**	**41**	**40**	**38**	**37**	**36**	**35**	**34**	**33**	**32**	**31**	**30**	**29**	**28**	**28**	**27**	**26**	**26**	**25**
205	(93.0)	46	44	43	41	40	39	37	36	35	34	33	32	31	30	29	29	28	27	26	26	25	24
**200**	**(90.7)**	**45**	**43**	**42**	**40**	**39**	**38**	**37**	**35**	**34**	**33**	**32**	**31**	**30**	**30**	**29**	**28**	**27**	**26**	**26**	**25**	**24**	**24**
195	(88.5)	44	42	41	39	38	37	36	35	33	32	31	31	30	29	28	27	26	26	25	24	24	23
**190**	**(86.2)**	**43**	**41**	**40**	**38**	**37**	**36**	**35**	**34**	**33**	**32**	**31**	**30**	**29**	**28**	**27**	**26**	**26**	**25**	**24**	**24**	**23**	**23**
185	(83.9)	41	40	39	37	36	35	34	33	32	31	30	29	28	27	27	26	25	24	24	23	23	22
**180**	**(81.6)**	**40**	**39**	**38**	**36**	**35**	**34**	**33**	**32**	**31**	**30**	**29**	**28**	**27**	**27**	**26**	**25**	**24**	**24**	**23**	**22**	**22**	**21**
175	(79.4)	39	38	37	35	34	33	32	31	30	29	28	27	27	26	25	24	24	23	22	22	21	21
**170**	**(77.1)**	**38**	**37**	**36**	**34**	**33**	**32**	**31**	**30**	**29**	**28**	**27**	**27**	**26**	**25**	**24**	**24**	**23**	**22**	**22**	**21**	**21**	**20**
165	(74.8)	37	36	34	33	32	31	30	29	28	27	27	26	25	24	24	23	22	22	21	21	20	20
**160**	**(72.6)**	**36**	**35**	**33**	**32**	**31**	**30**	**29**	**28**	**27**	**27**	**26**	**25**	**24**	**24**	**23**	**22**	**22**	**21**	**21**	**20**	**19**	**19**
155	(70.3)	35	34	32	31	30	29	28	27	27	26	25	24	24	23	22	22	21	20	20	19	19	18
**150**	**(68.0)**	**34**	**32**	**31**	**30**	**29**	**28**	**27**	**27**	**26**	**25**	**24**	**23**	**23**	**22**	**22**	**21**	**20**	**20**	**19**	**19**	**18**	**18**
145	(65.8)	33	31	30	29	28	27	27	26	25	24	23	23	22	21	21	20	20	19	19	18	18	17
**140**	**(63.5)**	**31**	**30**	**29**	**28**	**27**	**26**	**26**	**25**	**24**	**23**	**23**	**22**	**21**	**21**	**20**	**20**	**19**	**18**	**18**	**17**	**17**	**17**
135	(61.2)	30	29	28	27	26	26	25	24	23	22	22	21	21	20	19	19	18	18	17	17	16	16
**130**	**(59.0)**	**29**	**28**	**27**	**26**	**25**	**25**	**24**	**23**	**22**	**22**	**21**	**20**	**20**	**19**	**19**	**18**	**18**	**17**	**17**	**16**	**16**	**15**
125	(56.7)	28	27	26	25	24	24	23	22	21	21	20	20	19	18	18	17	17	16	16	16	15	15
**120**	**(54.4)**	**27**	**26**	**25**	**24**	**23**	**23**	**22**	**21**	**21**	**20**	**19**	**19**	**18**	**18**	**17**	**17**	**16**	**16**	**15**	**15**	**15**	**14**
115	(52.2)	26	25	24	23	22	22	21	20	20	19	19	18	17	17	16	16	16	15	15	14	14	14
**110**	**(49.9)**	**25**	**24**	**23**	**22**	**21**	**21**	**20**	**19**	**19**	**18**	**18**	**17**	**17**	**16**	**16**	**15**	**15**	**15**	**14**	**14**	**13**	**13**
105	(47.6)	24	23	22	21	21	20	19	19	18	17	17	16	16	16	15	15	14	14	13	13	13	12
**100**	**(45.4)**	**22**	**22**	**21**	**20**	**20**	**19**	**18**	**18**	**17**	**17**	**16**	**16**	**15**	**15**	**14**	**14**	**14**	**13**	**13**	**12**	**12**	**12**
95	(43.1)	21	21	20	19	19	18	17	17	16	16	15	15	14	14	14	13	13	13	12	12	12	11
**90**	**(40.8)**	**20**	**19**	**19**	**18**	**18**	**17**	**16**	**16**	**15**	**15**	**15**	**14**	**14**	**13**	**13**	**13**	**12**	**12**	**12**	**11**	**11**	**11**
85	(38.6)	19	18	18	17	17	16	16	15	15	14	14	13	13	13	12	12	12	11	11	11	10	10
**80**	**(36.3)**	**18**	**17**	**17**	**16**	**16**	**15**	**15**	**14**	**14**	**13**	**13**	**13**	**12**	**12**	**11**	**11**	**11**	**11**	**10**	**10**	**10**	**9**

Note: BMI values rounded to the nearest whole number. BMI categories based on CDC (Centers for Disease Control and Prevention) criteria.

www.vertex42.com BMI = Weight[kg] / (Height[m] x Height[m]) = 703 x Weight[lb] / (Height[in] x Height[in]) © 2009 Vertex42 LLC

References

1. EDLIZ 2015, 7th Essential Medicines List and Standard Treatment Guidelines for Zimbabwe
2. The British National Institute for Health and Care Excellence (NICE) guidance, Type 2 diabetes in adults: management, May 2017. *nice.org.uk/guidance/ng28*
3. NICE guidance, Hypertension in adults: diagnosis and management, November 2016. *www.nice.org.uk/guidance/cg127*
4. British National Formulary (BNF) 73, March-September 2017
5. Screening for type 2 diabetes mellitus, David K McCulloch, Rodney A Hayward, https://www.uptodate.com/contents/screening-for-type-2-diabetes-mellitus?search=screening%20for%20diabetes&source=search_result&selectedTitle=1~150&usage_type=default&display_rank=1 (accessed 15 Feb 2018)
6. Clinical presentation and diagnosis of diabetes mellitus in adults, David K McCulloch, https://www.uptodate.com/contents/clinical-presentation-and-diagnosis-of-diabetes-mellitus-in-adults?source=history_widget (accessed 15 Feb 2018)
7. 2019 ACC/AHA Guideline on the Primary Prevention of Cardiovascular Disease: A Report of the American College of Cardiology/American Heart Association Task Force on Clinical Practice Guidelines. *J Am Coll Cardiol* 2019;March 17:[Epub ahead of print].

MOHCC-MSF NCD PILOT
MANICALAND PROVINCE
ZIMBABWE
